# Development of the Mata Hari Judas Queen (*Felis catus*)

**DOI:** 10.3390/ani10101843

**Published:** 2020-10-10

**Authors:** Peter J. Murray, Melanie Rogie, Natalie Fraser, Julia Hoy, Samantha Kempster

**Affiliations:** 1 School of Sciences, Faculty of Health, Engineering and Sciences, University of Southern Queensland, Toowoomba QLD 4350, Australia; 2 School of Agriculture and Food Sciences, The University of Queensland Gatton campus, Gatton QLD 4343, Australia; melanie.mills@uqconnect.edu.au; 3 School of Veterinary Science, The University of Queensland Gatton campus, Gatton QLD 4343, Australia; natalie.fraser@uq.edu.au (N.F.); s.kempster@uq.edu.au (S.K.); 4 Hidden Vale Wildlife Centre, The University of Queensland, Grandchester QLD 4340, Australia; j.hoy@uq.edu.au

**Keywords:** compudose, oestrus, oestrous, feral cat, Judas, Mata Hari, queen, conservation

## Abstract

**Simple Summary:**

Predation by introduced feral cats is one of the main drivers of extinction in Australian mammals and they have been implicated in reducing populations of birds, frogs and reptiles. Current control techniques e.g., fencing, baiting, trapping and shooting for the management of cats are costly, labour intensive and fail to eradicate entire populations which allows survivors to re-establish populations. The Mata Hari Judas (MHJ) technique i.e., inducing prolonged oestrus using hormone implants, can enhance the eradication of remnant feral animals after the majority of their population has been killed. The hypotheses tested in this study were that hormone implants could induce prolonged oestrus in queens (adult female cats), and that prolonging oestrus resulted in sustained attractiveness to toms (adult male cats). This study shows that it is possible to induce and prolong oestrus in queens using hormone implants where these queens are attractive to toms. The MHJ queen is a new tool with the potential to enhance detection and thus the control of feral cats in remnant populations.

**Abstract:**

Cats (*Felis catus*) are significant predators of mammals, birds, frogs and reptiles and are implicated in mammal species extinctions in Australia. Current controls fail to eradicate entire populations allowing survivors to re-establish. The use of the Mata Hari Judas (MHJ) technique, i.e., inducing prolonged oestrus using hormone implants, can enhance the eradication of remnant animals and would greatly improve conservation efforts. The hypotheses tested were that hormone implants could induce prolonged oestrus in queens (adult female cats), and that prolonging oestrus would result in sustained attractiveness to toms (adult male cats). Queens (*n* = 14) were randomly allocated to five treatments including a control and four treatments using hormone implants. Queens were observed daily; alone and during indirect contact with a tom for 30 consecutive days. There were significant increases (*p* < 0.001) in oestrus duration (19 to 27 days) for entire and ovariohysterectomised queens given Compudose100™ implants (1/8 or 1/4 implant). This study shows that it is possible to induce and prolong oestrus in queens using Compudose100™ implants where these queens are attractive to toms. The MHJ queen is a new tool with the potential to enhance the detection and thus the control of feral cats in remnant populations.

## 1. Introduction

The introduced cat (*Felis catus*) occupies most of Australia and they are significant and major predators of native mammals, birds, frogs and reptiles and are implicated in the extinction of a number of native mammal species [1,2]. Current control techniques for the management of feral, stray and domestic cats are costly, labour intensive and fail to eradicate entire populations [3,4]. Although control techniques include baiting/poisoning (such as Eradicat^®^ or Curiosity^®^), lures, predator proof fencing, trapping, shooting and reproductive manipulation, they are largely unsuccessful in eradicating remnant populations of feral cats [4,5,6,7]. Survivors of these techniques are capable of re-establishing populations of feral cats. In countries such as Australia, where native animals have evolved without feline predators, populations of feral cats have drastic effects on native wildlife and thus must be completely removed if at all possible.

For gregarious species, the ‘Judas’ technique is a very effective method which involves capturing a wild animal in its usual territory, attaching a radio-tracking collar, then releasing the animal in the same location in anticipation that it will actively re-join or seek out remnant populations of its kind [8,9]. This technique is designed to be used as a complimentary method in collaboration with large scale control operations to eliminate remnant populations after mass eradication has been attempted [8,9,10,11]. The Judas technique was enhanced by Dr Karl Campbell when he developed the ‘Mata Hari Judas’ (MHJ) goat [12,13]. This involved improving the Judas technique by manipulating the reproductive system (surgically and hormonally) to sterilise and terminate pregnancy in female goats in a way that still permitted natural displays of sexual behaviours to increase the efficacy of the method [12,13,14]. The MHJ technique not only targets the gregarious nature of the species but also specifically targets the instinctual desire to mate, without the risk of reproduction [12,13].

The MHJ technique has only previously been used in gregarious species. This is a characteristic that cats lack as they are habitually solitary, highly territorial, and not gregarious [15,16]. However, the MHJ technique relies on the sexual behaviour of the species and cats are a highly promiscuous species. Therefore, after mass eradication attempts, remnant populations can rapidly repopulate that area. The demonstrated success of the MHJ technique in other species creates the potential for its use to detect and thus control feral cats.

The female cat oestrous cycle is highly variable, however, and the average cycle of a non-mated queen is typically 17 to 21 days [17,18,19]. The oestrous cycle of a non-mated queen, or when ovulation is not induced, consists of proestrus, oestrus, and interoestrus/postoestrus and the cycle repeats until ovulation or pregnancy occurs [17,20,21,22]. Proestrus has a mean of 2 days with a range of 1–2 days; oestrus has a mean of 7 days with a range of 2–19 days and interoestrus has a mean of nine days ranging from 2 to 17 days [18,20,22,23]. During oestrus, the queen displays obvious behavioural signals as this is the period of peak oestradiol (E_2_) production, the peak in follicular activity and she is receptive to mating [17,19,20].

To confirm the development of a Mata Hari Judas queen, that is attractive to toms, it is necessary to house queens and toms to record their behaviour. However, cats kept in inadequate housing, stressful or long-term confinement can begin to display behavioural problems that the owners or caregivers deem ‘unnatural’ and undesirable [24,25,26]. These behaviours can often be a stress-induced response by the cat due to an inadequate environment or the inability to adapt to confinement and express natural behaviours [24,25,27]. Behavioural problems associated with cats in confinement include aggression, inappropriate elimination, anxiety, fear, eating disorders, change in temperament and excessive grooming or vocalisation [25,26]. To minimise these behavioural problems, cats need to be kept in the best possible conditions including separate housing and appropriate enrichment.

The aims of this research were to: (1) determine the best method to put a queen into prolonged oestrus; (2) confirm that the treated queen is attractive to toms and the queen displays typical oestrus behaviours; (3) determine the duration of the continued oestrus by observing behaviour in the queen and how long she remains attractive to toms; and (4) determine any adverse physiological, behavioural or psychological effects on the queen from inducing and prolonging oestrus.

## 2. Materials and Methods

### 2.1. Study Area

This study was conducted within the Clinical Studies Centre (CSC) at the University of Queensland, Gatton Campus, in southeast Queensland, Australia. The queens were housed individually in cat condos within a separate ward used only for the queens in the research. The toms were individually housed in cat condos in a separate ward away from the queens and all other cats.

In the wards, the temperature was set at a constant 21 °C, with artificial fluorescent lighting that provided a day length of 14 h duration to most closely represent Australian summer photoperiods as queens are polyoestrous long-day breeders.

### 2.2. The Cats

This project was conducted with the University of Queensland Animal Ethics approval SAFS/413/16. All animal experiments and procedures were conducted to comply with the Australian Code for the care and use of animals for scientific purposes (EA28). This research had approval (Permit 7250) for the use of hormonal growth promotants in a non-bovine and non-bubaline animal by the Australian Pesticides and Veterinary Medicines Authority to comply with the Agricultural and Veterinary Chemicals Code Regulations (1995).

The cats were sourced from the Toowoomba Animal Management facility (TAM) within Toowoomba Regional Council (TRC) and Animal Management Centre within the Lockyer Valley Regional Shire Council, located in southeast Queensland, Australia. The facilities were located within approximately 50 km from the CSC. These cats were a combination of feral cats, stray and unclaimed domestic cats that had, from their behaviour, some experience with humans.

Cats spent at least a week in the respective animal management facilities and were selected for this study on the basis that they were not obviously pregnant, visually in reasonable health (e.g., no injuries or missing hair) and the staff were reasonably able to handle the cats i.e., the cats were not overly aggressive. It took two months to acquire sufficient female cats for the study, as on average the facilities only had two suitable cats available each week. The toms were selected on the same basis, and that they were visibly intact.

After transport to the CSC, all cats were isolated for a minimum of 10 days to observe for signs of illness or disease, and to monitor general health and wellbeing. After the isolation period, the cats were placed in their condo until sufficient cats were available to start the experiment. Consequently, most cats were resident in the condos for several months prior to the start of the experiment and well adjusted to the CSC with regular handling by humans.

All queens (*n* = 14) were entire, and ultrasound was used to determine if any were pregnant upon arrival at the CSC. Early pregnancy was confirmed via ultrasound in two queens that were treated with aglepristone (10 mg/kg, intramuscular injection (IM), twice 24 h apart) to induce abortion prior to the start of the study [28,29]. Treatment with aglepristone was given 113 days prior to the start of behavioural observations, and neither queen displayed adverse effects after abortion was induced.

Ages for all cats were estimated based on their sex, breed, size, dentition, weight and reproductive status. At the start of the experiment, the queens were estimated to range from 7 months to 4 years old. The queens were a mix of domestic short hair (DSH) and domestic long hair (DLH) breeds and had a weight range of 2.8–4.1 kg. Both toms were DSH breeds and estimated to be 5–7 years of age with intact developed testicles and penile spines on their glans penis.

### 2.3. Treatments and Procedures for Queens

The queens were randomly allocated to 1 of 5 treatments: (A) untreated control; (B) Compudose100™ 1/8 implant, approximately 2.625 mg oestradiol 17β; (C) Compudose100™ 1/4 implant, approximately 5.25 mg oestradiol 17β; (D) Suprelorin^®^ 4.7 mg deslorelin; and (E) ovariohysterectomy + Compudose100™ 1/8 implant, approximately 2.625 mg oestradiol 17β. Where necessary, implants were cut with a sterile scalpel blade to achieve the required size and dosage rate. Confirmation was obtained from the manufacturer that the implants were a homogenous mix to confirm cutting the implant would achieve the desired dosage rate.

There were two replicates of treatments as the CSC could only have 10 female cats in a ward and for the second replicate, we were unable to source 10 suitable female cats. Therefore, two control queens from replicate 1 (R1) were recycled into replicate 2 (R2), and randomly re-allocated into a new treatment.

R1 (*n* = 10) had two queens assigned to each treatment. R2 (*n* = 6) had one queen assigned to each treatment with the exception of Treatment E, which had 2 queens. There was an additional queen allocated into Treatment E for R2 as this queen had undergone ovariohysterectomy approximately 12 weeks prior to R2. The purpose for this was to determine if oestrus could be induced in a queen that had been reproductively inactive for a longer period. It was also to ensure that there was no inhibition or feedback from the normally circulating (or declining) hormones compared to a recently spayed queen. Mean weight of queens for each treatment group was: A 3.07, B 3.03, C 2.83, D 3.5 and E 3.18 kg.

Queens in the control treatment (A) had no manipulation of their natural oestrous cycle and were not implanted with a pseudo-implant. The status of the oestrous cycle of the cats was not determined using vaginal swabs as these are to known to induce/trigger spontaneous ovulation (thus interfering with treatments by bringing the cat into a phase of dioestrus) and would have been stressful for the cats, as would have regular blood sampling.

Queens in Treatments B and C received sedation with dexmedetomidine and a local subcutaneous injection of lignocaine (1–2 mg/kg) prior to surgery. A rectangular area (approximately 4 × 3 cm) of the dorsal shoulder hair was clipped and the implantation site at the periscapular region was disinfected with chlorhexidine and methyl alcohol. The Compudose 1/8 implant was inserted subcutaneously via a 1 cm incision and the incision site secured. Atipamezole was used to reverse the sedation after the procedure was completed. Each queen received meloxicam (0.3 mg/kg) for post-implantation analgesia.

Queens in Treatment D did not require sedation, analgesia or surgical implantation for the implant. The implants were administered in the periscapular region using the manufacturer provided implantation device. Queens in Treatment E underwent general anaesthesia per standard protocol for feline ovariohysterectomy. The Compudose 1/8 implant was inserted and secured as per Treatments B and C.

All cats were fed a commercially available diet to meet maintenance energy requirements. The cats were fed using a combination of bowl and scatter feeding and supplemented with enrichment foods to provide mental stimulus and encourage natural behaviours. Water bowls provided ad libitum access to fresh water.

### 2.4. Observations of Behavioural Oestrus

Queens were observed for 30 consecutive days in each replicate for behavioural oestrus starting three days after the treatment procedures had been completed. R1 was conducted from 2 May 2017 to 31 May 2017. R2 was conducted from 10 June 2017 to 9 July 2017. To ensure that the queens displayed the same behaviours regardless of the presence or absence of the researcher and during different times of the day, two observation methods were used: in-ward physical assessment and continuous video monitoring for displays of behavioural oestrus.

#### In-Ward Physical Assessment for Display of Oestrus Behaviours

The sequence of in-ward physical assessments for each queen was randomly selected daily. Each queen was removed from her condo and permitted to roam freely within the housing ward. The queen was observed and assessed based on nine of the common oestrus behaviours. Every day an ‘oestrus score’ was measured on a 0 to 9 scale, for each queen, for each of the oestrus behaviours. A queen was considered in oestrus when the score was 5 or above. Oestrus duration was measured as the number of consecutive days where the oestrus score was 5 or above. The nine behaviours observed for the oestrus score are defined in Table 1.

Continuous video monitoring was conducted of each queen inside the condo. There were five cameras installed in the queen ward, with each individual camera observing two condos. The video observations of the queens were not scored but used to confirm the in-ward oestrus behaviour assessment scores while the cats were in the condos and during dark photoperiods. Video observations were also observed to determine if any queens displayed any adverse behavioural or physiological events that were not observed during physical assessments.

### 2.5. Monitoring Cat Health and Adverse Behaviours

Each day a health and wellbeing diary was completed for each cat by the husbandry staff. This diary recorded and monitored food and water intake, feeding method, urine and faeces (consistency and volume), enrichment activity and time, medical or behavioural issues and if treatment was required. The cats were weighed weekly, which was recorded in the diary and monitored for any significant change.

The behaviour of all cats was examined daily during physical assessments, general care by staff, video footage and updating the daily diary for each cat. If any medical or behavioural issues were diagnosed or identified, further medication and treatment was provided and recorded.

### 2.6. Queen and Tom Behaviours

To determine the duration of oestrus in queens their behaviours and how long they remained attractive to the toms we used three cages that allowed the introduction of the queen to the tom without permitting direct contact. The cages were steel transport crates that measured 845 mm high by 1210 mm wide by 755 mm deep, with bar spacing 33 mm wide. The cages were set up in the feline isolation ward for introductions, the same ward in which the toms were housed.

Cage 1 was only used for tom 1 for the duration of the project, similarly, cage 2 for tom 2. Cage 3 was used for all queens during introductions. The cages were placed at ground level, with cage 3 for the queens positioned in-between the tom cages 1 and 2, and a 28 cm space either side of each cage. Once the first tom was inside the cage, a queen was placed into the centre cage. The handler would leave the room allowing the tom and queen to interact for up to 10 min. The handler observed and recorded oestrus and courtship behaviours of the queen and tom through a viewing window.

The sequence of introductions for both the queens and the toms was randomly allocated for each introduction day. Introduction days and times were randomised throughout each 30 day replicate. R1 had 16 introduction days and R2 had 12 introduction days. Tom and queen introductions were not conducted daily to allow the cats a rest period. As the introduction room was also the same ward in which the toms were normally housed, sheets were placed over the doors and windows of the toms housing condos to obscure the view to the tom not being used during introductions.

Once secured inside the cage, the queen was observed and assessed based on nine of the common oestrus behaviours (Table 1). The only difference to the nine parameters observed during introductions was the removal of ‘vaginal secretion’, which was replaced with ‘backing-up to the tom’. The queen was considered as ‘backing-up to the tom’ when she would push her hind quarters up against the cage when the tom was in the same area, this was often accompanied with lordosis. An ‘oestrus score’ was again measured on a 0–9 scale on each introduction, for each queen. A queen was considered as displaying typical oestrus behaviours in front of the tom when the score was 5 or above. During the 10 min test period, if the queen scored 7 or above, the observations for that queen ceased.

After the first observation period was complete, the first tom was removed and placed back into his condo. The queen remained in the centre cage. Once the second tom was in his introduction cage, the entire process and observations were repeated.

During the introductions to the queens, the tom was observed and assessed based on 7 common courtship behaviours. A ‘courtship score’ was measured on a 0–7 scale during each introduction, for each tom (Table 2). An overall score was then given for each tom on each introduction, 1 = interested (the courtship score was 4 or above) and 0 = not interested (the courtship score was 3 or below).

### 2.7. Statistical Analyses

General linear models were used to compare the duration of oestrus in days and the average oestrus scores for each treatment group. A random effect was added to code for queen identity to account for a lack of independence due to the repeated measures in queens [38]. The main fixed effect of interest was the Treatment with 6 levels: pre-treatment observation, A, B, C, D and E. A fixed effect coding for cycle was added to the model assessing the oestrus score. Data were confounded by factors including cat illness or injury, euthanasia and environmental conditions which resulted in incomplete data sets.

Marginal means were estimated as follow-up estimates derived from the multivariable models. Pairwise comparisons were used to compare the marginal means for each treatment group. *p*-values from the pairwise comparisons were adjusted for multiple comparisons using the false discovery rate approach described by Pike (2011) [38]. All analyses were conducted in Stata (www.stata.com) using an alpha of 0.05, and only *p* values < 0.05 were considered significant.

## 3. Results

### 3.1. Observations of Oestrus in the Queen

The marginal means of oestrus duration (days) were significantly increased in Treatments B, C and E, with marginal means of 27.1, 24.2 and 19.2 days, respectively (*p* < 0.001), compared to the pre-treatment mean of 2.9 days (Table 3). The marginal means of Treatments A and D were not significantly different (*p* > 0.05) to the pre-treatment mean.

**Figure 1:**
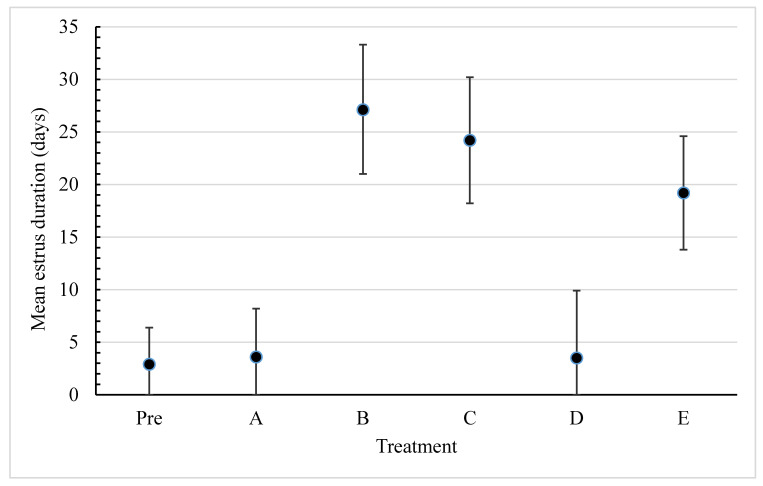
Marginal mean oestrus duration (days) for queens in each treatment, and the pre-treatment observation period. Bars represent 95% confidence intervals.

The results demonstrate that the marginal mean oestrus duration was prolonged in Treatments B, C and E compared to all other Treatments and pre-treatment observations (Figure 1).

Queen 8450, in Treatment E, demonstrated that behavioural oestrus could be induced using Compudose100™ at a dosage rate of 2.625 mg oestradiol 17β, even though this queen had undergone ovariohysterectomy 12 weeks prior to the hormonal implant. During the 12 week pre-treatment period, this queen displayed no behavioural signs of oestrus and had a mean oestrus score of 0. However, during observations for behavioural oestrus in Treatment E, this queen had a mean oestrus score of 7.9 (on a scale from 0 to 9) and her mean oestrus duration (days) was 30 days. This meant that Queen 8450 was able to be successfully induced into behavioural oestrus and displayed continued oestrus for the full 30 days of in-ward observations.

### 3.2. Queen and Tom Behaviours

The exposure of the queens to toms was based on the observed behaviour for the same queens on the same day when exposed to one of the two toms (separate exposures separated by time but occurring on the same day). Data were limited to those queens that were showing oestrus (score 5 or greater), and for which there were complete data (queens had all three observations on all days: oestrus behaviour in the presence of tom 1, and then separately, behaviour in the presence of tom 2) (Table 4).

Queens that were observed in oestrus in Treatment A only displayed oestrus behaviours in the presence of both tom 1 and tom 2 for 16.0% of all interactions. In contrast, the queens that were observed in oestrus in Treatment E displayed oestrus behaviours in the presence of tom 1 and tom 2 for 84.8% and 76.1% of interactions, respectively.

There was stronger agreement for the observed behaviour for those queens that were not showing any signs of behavioural oestrus when observed on their own. On all occasions where these queens were exposed to a tom, the observed behaviour was the same (no signs of oestrus).

In a similar manner, the queens that did not have observations consistent with oestrus behaviour did not show oestrus behaviour in the presence of a tom. The number of days and the average of the number of attraction days (the number of days when a tom was observed to be attracted to the queen) when the queen was confirmed to be in oestrus are given in Table 5.

There was a total of 25 days where queens in Treatment A were in oestrus when introduced to the toms (Table 5). Tom 1 showed attraction in 24% of interactions, and tom 2 showed attraction in 16% of interactions. In contrast, there were 48 days where queens in Treatment E were in oestrus when introduced to the toms. Tom 1 showed attraction in 70.8% of interactions, and tom 2 showed attraction on 43.7% of interactions. These results across all treatments show that tom 1 was more responsive and displayed higher attraction to the queens in Treatments A, D and E. Tom 1 and tom 2 showed equal attraction to the queens in Treatments B and C.

The ‘average attraction days’ was the rounded average of the two separate values (one for each tom) and also expressed as a percentage of attraction days. Using these averages, the toms displayed greater attraction to queens in Treatment C (58.8% attraction days), followed by Treatment E (58.3% attraction days), and Treatment B (40% attraction days).

There was a total of 59 days when the queens were observed with oestrus scores between 0 and 4 (not in oestrus) and were then exposed to toms. There were only two occasions when a tom was attracted to a queen who was displaying an oestrus score between 0 and 4. Both of these instances involved tom 2 and one involved a queen in treatment A and the other a queen in treatment E.

### 3.3. Cat Health and Adverse Behaviours

**Figure 2:**
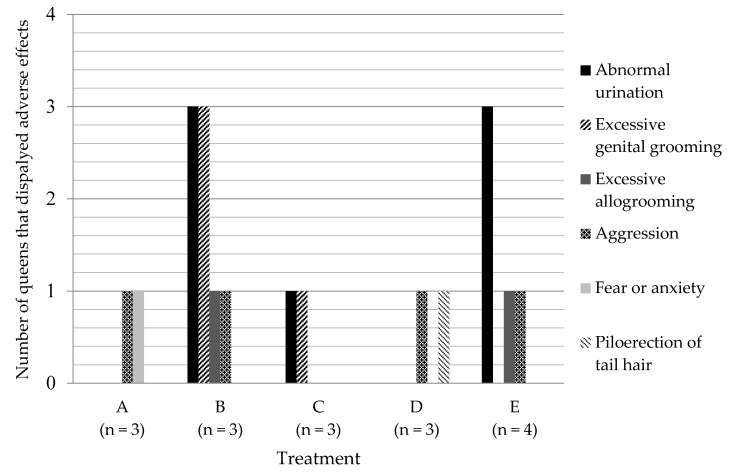
The number of queens (*n*) that displayed different adverse effects in each treatment.

A number of adverse effects were identified and these included abnormal urination (e.g., cats urinating outside the litter tray and impulsive elimination), although this began and increased in frequency during R2 following a change in the soft litter substrate to use of Catrine balls to collect the queens urine as part of a different research project, excessive genital grooming, excessive allogrooming, aggression, fear or anxiety and the persistent piloerection of the tail hair (Figure 2). Fear or anxiety was determined by monitoring behavioural changes such as hissing, crouching with ears pinned back, avoidance, and retreating from handlers or other cats, trembling, flinching and dilated pupils.

Treatment B had queens with the greatest number of adverse effects (Figure 2). All queens in this treatment displayed both abnormal urination and excessive genital grooming, and 33.3% displayed both excessive allogrooming and aggression. All queens in Treatment B were negatively affected and displayed at least two adverse effects per queen. One queen (8190) from Treatment B was euthanized at the completion of R1. This queen had been showing aggression, avoidance behaviours, along with abnormal urination.

Only one queen in Treatment C displayed adverse effects that were abnormal urination and excessive genital grooming. In Treatment D, only one queen displayed adverse effects that were aggression and piloerection of the tail. In Treatment E, 75% of queens displayed abnormal urination. One queen additionally displayed excessive genital grooming, and another queen displayed aggression. All queens in Treatment E were negatively affected and displayed at least one adverse effect per queen. One queen (8303) was required to be euthanized on the 11^th^ day of repeat 2 due to significant and increasing aggression.

Abnormal urination was the most significant adverse effect observed, but was only observed in Treatments B, C and E. In Treatments B, C and E combined, 70% of queens displayed frequent abnormal urination.

The two queens that required euthanasia (8190 and 8303) were sent to the University of Queensland, School of Veterinary Science Pathobiology Laboratory for full necropsy. Queen 8190 had significant thickening (approximately two-fold) in a segmental pattern of the uterus, but no obvious corpus luteum or follicles in the ovaries [26]. The endometrial layer was increased up to 2 mm thick by endometrial glands, there was abundant stroma and increased numbers of small calibre blood vessels, indicating endometrial hyperplasia associated with hypoestrogenism [26]. There was no obvious cause for the increased aggression in cat 8303. However, the cells of her zona fasciculata were expanded up to three-fold by clear discrete cytoplasmic vacuoles, indicating adrenocortical hypertrophy [39], also associated with hypoestrogenism as adrenocortical hypertrophy has been reported in other species in response to exogenous oestrogens [39].

Upon the completion of the experiment, the queens that were expected to be re-homed underwent ovariohysterectomy (Treatments A, B, C and D) and Compudose™ implants were removed (Treatments B and C). Similar to the pathology report for cat 8190, all queens that underwent ovariohysterectomy after the trial that were in Treatments B and C also had significant thickening in a segmental pattern of the uterus, and no obvious corpus luteum or follicles.

## 4. Discussion

This research demonstrated that it was possible to both induce and prolong behavioural oestrus in entire and ovariohysterectomized queens using hormonal implants. Highly significant increases (*p* < 0.001) in oestrus duration in queens in Treatments B, C and E, i.e., 27.1, 24.2 and 19.2 days, respectively, out of a maximum of 30 days, were all increased compared to the pre-treatment mean of 2.9 days and above the generally accepted mean of 7 days (range of 2 to 9 days) [18,19].

The results demonstrated that there was no significant difference in mean oestrus duration for Treatments A and D (*p* > 0.05) compared to the pre-treatment mean. All three queens in Treatment D had the initial oestrus induction (“flare-up effect”) that is typical for deslorelin treatment [40,41]. This was observed for 3, 6 and 12 days, for each of the three queens, respectively. However, their oestrus scores during in-ward assessments often only just met the minimum score of 5, and their oestrus behaviour was not prolonged.

Factors were identified that may have impacted on the behavioural displays of oestrus of the queen in the presence of a tom were that the queens were rapidly exposed to an environment with new unfamiliar scents (such as the toms), brighter fluorescent lighting and there were often interruptions from students or volunteers [31,42] that may have distracted both the queen and the tom during introductions.

Some queens were frequently observed showing signs of fear and anxiety once they were secured inside the queen cage in the tom housing ward for introductions. These included queens in a couched sitting position with the tail tucked under the body, her ears pinned back, and pupils enlarged, piloerection of the hair, shaking and increased displays of aggression [33].

There were days when queens displayed obvious behavioural oestrus during in-ward assessments, and in the presence of a tom, however, the tom was often not responsive. Toms showed greater attraction to queens in Treatment C, followed by Treatments E and B. These Treatments were all Compudose100™ at varying dosage rates, the same Treatments that had the highest efficacy for the queen to display behavioural oestrus during in-ward assessments.

### Adverse Effects on Queens

There were six adverse effects identified in the queens during this research. Not all of the adverse effects were seen in all queens, however, the majority of adverse effects, primarily abnormal urination, were observed in the Compudose-treated queens (Figure 2) and abnormal urination was likely due to hypoestrogenism from exogenous oestrogens from the Compudose100™ implants [26,39]. However, another reason for this abnormal urination may have been the change of litter substrate from soft absorbent litter to non-absorbent Catrine balls, as cats will often avoid areas that smell strongly of urine and often exhibit undesirable elimination [24,25,27,43]. As the Catrine balls do not absorb any of the moisture, it is highly likely that the cats avoided elimination in their trays.

Four of the 14 queens displayed aggressive behaviours. These queens were in different Treatments (A, B, D and E) and it is possible that the aggression was due to factors unrelated to the research, i.e., two of these queens had shown behavioural signs of aggression prior to the start of the research. One queen from Treatment D displayed piloerection of the tail hair, but the cause of this could not be established. This queen did not display this physiological effect prior to the treatment with Suprelorin^®^ but it was observed continuously post treatment.

Queens that were in any Compudose100™ treatment were either spayed post-experiment or underwent postmortem examination; all had significant abnormalities of the uterus and ovaries. To mitigate the risk in cats of developing adverse effects such as abnormal urination and abnormalities of the uterus and ovaries, we recommend that implants should only be used for several weeks.

## 5. Conclusions

The use of the Mata Hari Judas technique has been shown to be successful in the eradication of other pest species and has great potential to increase the efficiency and capability of feral cat control, particularly for eradicating remnant populations of cats after the majority of cats have been controlled. These results show that Compudose100™ can be used to induce and prolong oestrus behaviour in queens and future research should extend to field trials for developing methods of using MHJ queens in situ. These might include the development of lures using urine from MHJ queens to use with existing detection and control methods (e.g., the Felixer; [44]) or the release of radio-collared MHJ queens to find the remaining cats within an area after traditional methods have eradicated the majority of the cats, which has been critical for the success of eradication of other species e.g., goats [45].

## Figures and Tables

**Table 1:** Behaviours and vaginal secretion used to measure and score queens in oestrus.

Parameter	Definition	References
Calling	Prolonged or repeated vocalisation.	Jemmett and Evans (1977) [30]; Liberg (1983) [31]
Scent marking	The queen repeatedly rubbed/brushed her cheek and neck to mark her scent on objects.	Mellen (1993) [32]; Stanton et al. (2015) [33]
Treading	The queen displayed a rhythmic, raising and lowering of the hind legs so that she was stepping in place without locomotion.	Mellen (1993) [32]; da Silva et al. (2006) [17]; Stanton et al. (2015) [33]
Tail deflection	The queen moved her tail off to one side (also at a slightly upwards angle) in a stationary position; normally seen in conjunction with lordosis.	Mellen (1993) [32]; Stanton et al. (2015) [33]
Lordosis	The queen raised hindquarters while lowering the forequarters onto the ground, presenting genitals with her tail usually positioned to one side. This posture was stationary, however, it was sometimes accompanied by treading of the hind legs.	Stanton et al. (2015) [33]
Rolling	Whilst the queen was lying, her body rotated from one side to another and this action was repeated. When rolling her back was rubbing against ground, the belly exposed and all paws extended in the air.	Stanton et al. (2015) [33]
Flirt walk	The queen cat feigned walking/running back and forth or around the potential breeding partner.	Liberg (1983) [31]; Stanton et al. (2015) [33]
Genital groom	The queen groomed its own genitals itself by licking, biting or chewing the fur around the vaginal region. Often seen with increased vaginal secretion.	Stanton et al. (2015) [33]
Vaginal secretion	The secretion of a clear and viscous fluid was discharged from the vagina.	da Silva et al. (2006) [17]

**Table 2:** Tom courtship and mating behaviours in response to the presence of a queen in oestrus.

Behaviour	Definition	References
Calling	Prolonged or repeated vocalisation.	Jemmett and Evans (1977) [30]; Liberg (1983) [31]; Mellen (1993) [32]
Scent marking	The tom repeatedly rubbed/brushed his cheek and neck to mark his scent on objects and often on a potential mate.	Mellen (1993) [32]; Stanton et al. (2015) [33]
Spraying	When standing with the tail vertically erect, tom released a jet of urine backwards against a vertical surface or object.	Natoli and De Vito (1991) [34]; Mayes et al. (2015) [35]; Stanton et al. (2015) [33]
Flehmen	Tom made a scowl-like facial expression, mouth open, upper lip elevated and teeth often exposed. The tongue may protrude out of the mouth; the tom using olfactory senses to detect scents.	Mellen (1993) [32]; Stanton et al. (2015) [33]
Stalking	A very slow and controlled forward walk in a crouched position directed towards the target (queen in oestrus), the head kept low and eyes focused on the target.	Stanton et al. (2015) [33]
Aggression	Included: a) growling—a low pitched, throaty, deep rumbling vocalisation produced while mouth closed or teeth may be exposed, b) striking—swiping a forepaw but no direct contact, and c) attack—the tom launches itself at a target with extended forelegs and attempts to engage in physical conflict.	Natoli and De Vito (1991) [34]; Bradshaw et al. (2007) [36]; Yeon et al. (2011) [37]; Stanton et al. (2015) [33]
Tom attempts to mount cage	The tom attempted intromission by attempting to climb or straddle the cage with front and hind feet whilst the female was in close proximity. Often accompanied by a treading movements of the hind legs and thrusting actions with his pelvis against the cage.	da Silva et al. (2006) [17]; Stanton et al. (2015) [33]

**Table 3:** Marginal mean oestrus duration in days, derived from the multivariable model, se = standard error, CI = confidence interval. Mean oestrus length for the five treatments is out of a maximum of 30 days.

Treatment	Mean Oestrus Length (Days)		95% CI	
		se	Lower	Upper
**Pre**	2.9	1.8	0.0	6.4
**A**	3.6	2.3	0.0	8.2
**B**	27.1	3.1	21.0	33.3
**C**	24.2	3.1	18.2	30.2
**D**	3.5	3.3	0.0	9.9
**E**	19.2	2.8	13.8	24.6

**Table 4:** The total number of days where queens, within treatment groups, displayed signs of oestrus (oestrus score of 5 or greater) when they were observed with each of the toms. CI = confidence interval.

Treatment	Category	Estrus	No Oestrus	% In Oestrus	95% CI	
					Lower	Upper
Control (A)	Exposure to tom 1	4	21	16.0	6.4	34.7
Control (A)	Exposure to tom 2	4	21	16.0	6.4	34.7
B	Exposure to tom 1	15	15	50.0	33.2	66.8
B	Exposure to tom 2	18	12	60.0	42.3	75.4
C	Exposure to tom 1	21	13	61.8	45.0	76.1
C	Exposure to tom 2	22	12	64.7	47.9	78.5
D	Exposure to tom 1	1	8	11.1	2.0	43.5
D	Exposure to tom 2	1	8	11.1	2.0	43.5
E	Exposure to tom 1	39	7	84.8	68.1	89.8
E	Exposure to tom 2	35	11	76.1	59.0	83.4

**Table 5:** The total number of days when all queens in each treatment, during the 30 day treatment period, were observed in oestrus alone, and the number of days the two toms were attracted to these queens, and as a percentage of the total days queens were in oestrus.

Treatment	Queen Oestrus Days	Tom Attraction Days				95% CI	
	N	Tom 1	Tom 2	Average	%	Lower	Upper
A	25	6	4	5	20.0	8.9	39.1
B	30	12	12	12	40.0	24.6	57.7
C	34	20	20	20	58.8	42.2	73.6
D	9	3	1	2	22.2	6.3	54.7
E	48	34	21	28	58.3	44.3	71.2
